# Circumventing the scaling relationship on bimetallic monolayer electrocatalysts for selective CO_2_ reduction†

**DOI:** 10.1039/d2sc00135g

**Published:** 2022-03-14

**Authors:** Zhonglong Zhao, Gang Lu

**Affiliations:** School of Physical Science and Technology, Inner Mongolia University Hohhot 010021 China; Department of Physics and Astronomy, California State University Northridge California 91330 USA ganglu@csun.edu

## Abstract

Electrochemical conversion of CO_2_ into value-added chemicals continues to draw interest in renewable energy applications. Although many metal catalysts are active in the CO_2_ reduction reaction (CO_2_RR), their reactivity and selectivity are nonetheless hindered by the competing hydrogen evolution reaction (HER). The competition of the HER and CO_2_RR stems from the energy scaling relationship between their reaction intermediates. Herein, we predict that bimetallic monolayer electrocatalysts (BMEs) – a monolayer of transition metals on top of extended metal substrates – could produce dual-functional active sites that circumvent the scaling relationship between the adsorption energies of HER and CO_2_RR intermediates. The antibonding interaction between the adsorbed H and the metal substrate is revealed to be responsible for circumventing the scaling relationship. Based on extensive density functional theory (DFT) calculations, we identify 11 BMEs which are highly active and selective toward the formation of formic acid with a much suppressed HER. The H–substrate antibonding interaction also leads to superior CO_2_RR performance on monolayer-coated penta-twinned nanowires.

## Abstract 2

Dual-functional active sites are designed to circumvent the scaling relationship between the HER and CO_2_RR on bimetallic monolayer electrocatalysts.

## Graphical abstract

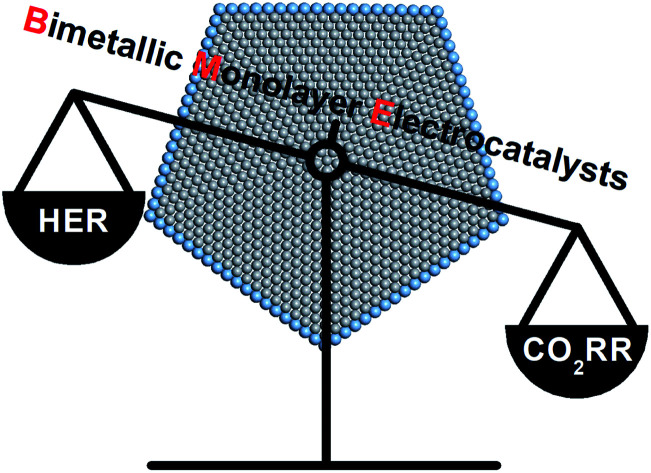

## Introduction

Electrochemical CO_2_ reduction reaction (CO_2_RR) yielding value-added chemicals and fuels *via* renewable energy sources, such as solar, hydro, and wind, has been recognized as one of the most attractive means of mitigating the pressing energy and environmental concerns.^1–3^ The daunting challenge, however, is to discover highly active, selective and stable catalysts for the CO_2_RR. Although CO_2_ can be electrochemically reduced to various products, including formic acid (HCOOH), carbon monoxide (CO), methane (CH_4_), and ethylene (C_2_H_4_) on transition metals (Cu, Au, Ag, Pd, *etc.*) and their alloys,^4–9^ the reaction kinetics and selectivity are rather low. For example, as one of the most studied metal catalysts for the CO_2_RR, Cu is capable of producing hydrocarbons, such as CH_4_ and C_2_H_4_. However, it does so with a substantial overpotential at ∼1 V *vs.* reversible hydrogen electrode (RHE) and with more than a dozen byproducts.^6,10^ Other metal catalysts also suffer from the same problems if not worse. Among the various culprits behind the poor activity and selectivity for the CO_2_RR, the facile hydrogen evolution reaction (HER) is believed to be the most detrimental. Highly active on metal surfaces and competing for protons with the CO_2_RR, the HER can significantly cut down the faradaic efficiency of the CO_2_RR.^11,12^ Thus, suppressing the HER becomes one of the most sought-after goals in developing metal catalysts for the CO_2_RR. This however turns out to be a difficult task as one must overcome the so-called energy scaling relationship between the two reactions.

As a central concept in the heterogeneous catalysis of transition metals, the energy scaling relationships refer to scaling correlations between surface bond energies of adsorbed species, including their intermediate and transition states.^13^ For example, *H and *COOH/*HCOO (* indicates adsorbed species) are intermediate species of the HER and CO_2_RR, respectively, and their binding energies on transition metal surfaces are found to be correlated with each other. The scaling relationships between *H and *COOH/*HCOO have been observed in pure metals,^14^ intermetallic alloys,^15^ and single-atom bimetallic alloys.^16^ In fact, it is believed that every imaginable active site structure would exhibit some scaling relationship between adsorption energies of various intermediates, one scaling relationship for each structure.^13^ Although the scaling relationships could be useful for understanding the trends and for fast screening of catalysts, they also impose severe limitations on catalyst development.^8,17^

It is generally recognized that the competition between the first hydrogenation reaction (CO_2_ + H^+^ + e^−^ → *COOH/*HCOO) and proton discharge (H^+^ + e^−^ → *H) is the most crucial step for the CO_2_RR, determining its overall efficiency and selectivity.^15,16^ To boost the hydrogenation reaction, one needs to strengthen the binding of *COOH/*HCOO on the surface. To hinder the proton discharge and *H evolution, on the other hand, one needs to weaken the binding of *H on the same surface. This poses a challenge since it violates the energy scaling relationships, which demand that the binding energies of *COOH/*HCOO and *H must increase or decrease simultaneously on the same reaction site.

Herein, we show that one can actually circumvent the scaling relationships by producing dual-functional sites on bimetallic monolayer electrocatalysts (BMEs), which consist of a transition metal monolayer (ML) on top of a transition metal substrate.^18,19^ We find that although the competing species, *H and *COOH/*HCOO, are anchored next to each other on the ML, *H binds to a threefold hollow site while *COOH/*HCOO binds to a top site. As a result, *H interacts strongly with the substrate while such an interaction is negligible for *COOH/*HCOO which bonds primarily to the ML. In other words, the BMEs realize dual-functional active sites for the HER and CO_2_RR which can circumvent the scaling relationships. In particular, one can engineer BMEs to modulate *H–substrate interactions and thus suppress the HER.

To elucidate the principles behind our design strategy, we first establish a linear scaling relationship between the adsorption free energies of *H and *COOH/*HCOO on 15 transition metals which form the basis of our investigation; the HER is shown to be facile on these metals. Next, taking these metals as the substrates, we demonstrate that *H adsorption free energies on the BMEs are altered significantly, and more importantly the linear scaling relationship is broken. As a result, 11 highly active and selective BMEs are identified for the CO_2_RR yielding HCOOH with a more suppressed HER. Based on crystal orbital Hamilton population (COHP) analysis, we show that *H–substrate antibonding interactions are responsible for weakened *H adsorption on the BMEs and for circumventing the scaling relationship. Finally, we generalize the finding to penta-twinned bimetallic nanowires in which the dual-functional sites also play a crucial role in the active and selective CO_2_RR to produce HCOOH.

## Computational methods

DFT calculations are carried out with the Vienna *Ab initio* Simulation Package (VASP).^20^ The revised Perdew–Burke–Ernzerhof (RPBE) exchange–correlation functional^21,22^ is used and the plane-wave energy cutoff is taken as 400 eV. Five transition metals Cu, Ag, Au, Pd, and Pt are chosen as the monolayer and are placed on top of 15 transition metal substrates of various crystal structures, including hcp (Ti, Zr, Ru, Hf, Re, Os), fcc (Rh, Pd, Ir, Pt), and bcc (V, Nb, Mo, Ta, W). The corresponding surface for each substrate is chosen as (0001) for hcp, (111) for fcc, and (110) for bcc, owing to their close-packed structures and stability. A four-atomic-layer slab with a 3 × 3 in-plane supercell is constructed with the adjacent slabs separated by a 15 Å vacuum in the normal direction. The bottom layer of the slab is fixed at the equilibrium bulk geometry while the remaining layers are allowed to fully relax. The Brillouin-zone is sampled with a 3 × 3 × 1 *k*-mesh according to the Monkhorst–Pack scheme^23^ and all atomic structures are optimized until the forces are less than 0.02 eV Å^−1^. The energy barriers for the formation of HCOOH are determined using the Climbing Image Nudged Elastic Band (CI-NEB) method.^24^*Ab initio* molecular dynamics (MD) simulations are performed using an NVT ensemble.^25–27^ A 6 × 6 in-plane supercell is constructed and the Brillouin-zone integration is restricted to the Γ point in the MD simulations. Molecular mechanics (MM) simulations are performed to relax the atomic geometry of the penta-twinned bimetallic ML nanowire (∼8.5 nm in diameter) using the EAM potentials.^28^

The stability of BMEs can be assessed by the formation energy (*E*_f_) and the segregation energy (*E*_seg_). *E*_f_ = (*E*^ML^_/sub_ − *E*_sub_ − *E*^ML^)/*N*, where *E*^ML^_/sub_ and *E*_sub_ represent the energy of a substrate with and without a coated metal ML, respectively. *E*^ML^ denotes the energy of the freestanding metal ML and N is the number of atoms in the slab model. *E*_sub_ is calculated as the energy of a four-atomic-layer substrate slab minus the energy of a freestanding substrate ML to balance the chemical equation. A BME is deemed stable if its *E*_f_ is lower than that of the reference, *i.e.*, Au^ML^/W(110), which is stable and has been experimentally synthesized.^29^*E*_seg_ is defined as the energy cost for swapping an atom in the ML with an atom in the substrate.^30^ The ML is considered stable if the segregation is endothermic (positive *E*_seg_). Based on *E*_f_ and *E*_seg_, 55 stable BMEs are identified (Table S1†) and are the subject of the following study. We further examine the stability of these 55 BMEs in the presence of key reaction intermediates, such as COOH* and HCOO* on their surfaces. The BME is deemed stable if no substantial surface reconstruction takes place after a full relaxation of the atomic geometry. *Ab initio* MD simulations are further performed to validate the structural stability of a subset of the BMEs (Fig. S1†). Finally, to evaluate the electrochemical stability of the BMEs, the dissolution potential *U*_diss_ is calculated.^31,32^*U*_diss_ is defined as *U*_diss_ = *U*_diss_(bulk) − *E*_f_/*eN*_e_, where *U*_diss_(bulk) is the standard bulk dissolution potential of the ML metal and *N*_e_ is the number of electrons involved in the dissolution. A positive *U*_diss_ suggests that the BME is electrochemically stable under acidic conditions (Table S2†).^32^ The possible oxidation of oxophilic substrates such as Mo and W is not considered here since the substrates are protected by the MLs. Our previous study also showed that Mo and W atoms buried inside bimetallenes could indeed retain their metallic states.^33^ Finally, we note that Pt and Pd MLs supported on Au, Ir, Pd, Rh, and Ru single crystals or nanostructured cores have been synthesized in experiments and found active for oxygen reduction, ammonia oxidation, formic acid oxidation, *etc.*^34–37^

The computational hydrogen electrode (CHE) model is used to estimate the free energy change (Δ*G*) at each intermediate step of the CO_2_RR and HER.^38^ In the CHE model, the chemical potential of a proton–electron pair, *μ*(H^+^ + e^−^), is defined in equilibrium with one half of the chemical potential of gaseous H_2_, *μ*(H_2_)/2, at 0 V, 101 325 Pa, and any pH values. When an external potential *U* is applied, *μ*(H^+^ + e^−^) is shifted by −*eU* (*e* is the elementary positive charge). The limiting potential (*U*_L_) for each intermediate step is defined as *U*_L_ = −Δ*G* at *U* = 0 V and the reaction overpotential (*U*_OP_) is the least *U*_L_ at which all intermediate steps are exergonic. To facilitate computational screening, we adopt an approximate solvation model to avoid highly expensive computation of an explicit solvation effect.^5,39,40^ To determine the relevant activation barriers, an ice-like water bilayer in a “H-down” configuration^41^ is placed on top of adsorbed reactants to simulate the solvation effect. Further details about the reaction models, free energy and activation energy calculations can be found in the ESI.†

## Results and discussion

**Fig. 1:**
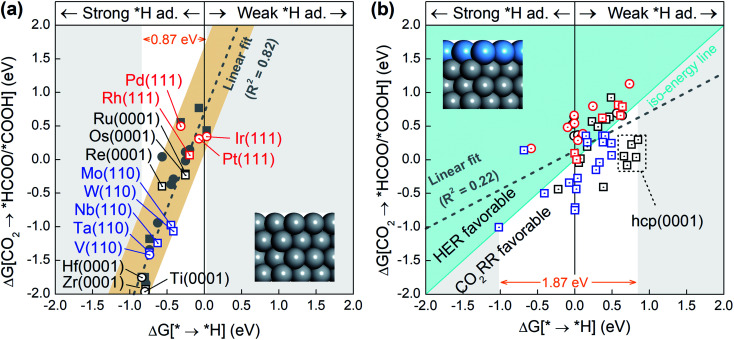
The change in free energy for the formation of the favorable intermediate, *COOH (circles) or *HCOO (squares), in the initial step of the CO_2_RR against the change in free energy for the formation of *H on (a) the pure metal substrate surfaces and (b) the 55 proposed BMEs. Linear fittings of the data points are shown as dotted gray lines. Iso-energy line for the formation of *H and *COOH/*HCOO on the BMEs is shown as a cyan solid line in (b). In the upper left of (b), the HER is more active, and in the lower right, the CO_2_RR is more active. The structural models for the pure metal substrates and BMEs are shown in the insets.

Since the competition between the CO_2_RR and HER is determined by the relative adsorption stability of the first hydrogenation intermediates, we compare in Fig. 1a and b the free energy of *H formation *versus* the free energy of CO_2_ reduction to *COOH/*HCOO on the proposed 15 metal substrates and 55 stable BMEs. As expected, the formation free energies of *H and *COOH/*HCOO on the pure metal substrates are linearly correlated with each other. The adjusted *R*-squared (*R*^2^) is 0.82 (Fig. 1a), suggesting that a linear energy scaling relationship holds on these metal substrates. Moreover, the HER is found facile on these substrates thanks to their modest *H formation energies (* → *H in Fig. 1a) with a narrow range (∼0.87 eV). To suppress the HER and boost the CO_2_RR, on the other hand, one needs to increase the formation energy of *H. To this end, we place a metal (Cu, Ag, Au, Pd, Pt) ML on top of these 15 substrates as shown in Fig. 1b (inset) to form 55 BMEs. Remarkably, the *R*-squared drops to *R*^2^ = 0.22 on these BMEs, indicating that the linear scaling relationship between the formation energy of *H and *COOH/*HCOO is broken. The broken scaling relationship would enable independent tuning of the HER and CO_2_RR on these BMEs, that is to say, one can increase the formation energy of *H (or weaken *H adsorption) and simultaneously decrease the formation energy of *COOH/*HCOO (or strengthen *COOH/*HCOO adsorption) on the BMEs to boost the CO_2_RR.

To rank the BMEs as potential catalysts toward the selective CO_2_RR, we compare the free energy change for *H formation *vs.* the free energy change for *COOH/*HCOO formation on each of the BMEs in Fig. 1b. The iso-energy line on which the two free energies are equal separates the BMEs into a HER favorable group and a CO_2_RR favorable group. 23 BMEs below the iso-energy line belong to the latter group and are more active toward the CO_2_RR than the HER as far as the first hydrogenation step is concerned. Interestingly, all 23 BMEs turn out to be selective in reducing CO_2_ to *HCOO, as opposed to *COOH, in the first step (squares in Fig. 1b). Note that the free energy span (1.87 eV) for *H formation on BMEs is much larger than that on the metal substrates (0.87 eV), suggesting that the substrates contribute significantly to *H adsorption on the BMEs.

**Fig. 2:**
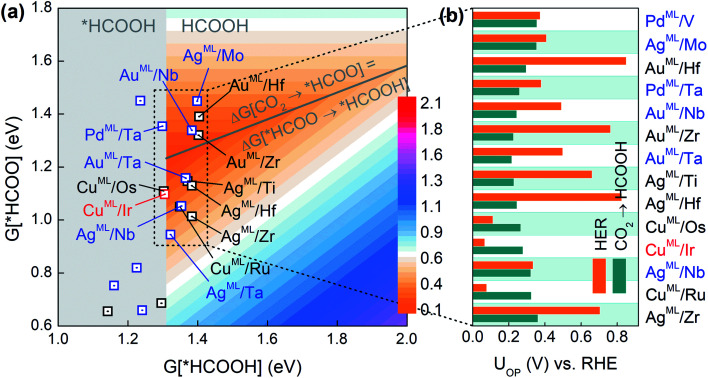
(a) Overpotential contour map toward HCOOH production in terms of the free energies of *HCOO and *HCOOH. (b) Comparison of *U*_OP_ for HCOOH production and the HER on selective BMEs.

As the second reduction step, *HCOO can be hydrogenated into *HCOOH with an additional proton transferred to the oxygen atom in *HCOO,^5^ and HCOOH would be the final product if the desorption of *HCOOH is exothermic. Based on the three-step reaction (CO_2_ → *HCOO → *HCOOH → HCOOH), we plot in Fig. 2a the overpotential (*U*_OP_) contour map for HCOOH production in terms of the free energies of *HCOO and *HCOOH. The contour map is constructed by calculating *U*_L_ for reaction steps CO_2_ → *HCOO and *HCOO → *HCOOH at each given *HCOOH free energy. The overpotential *U*_OP_ is defined as the larger *U*_L_ value between the two steps. Note that in any two-step reaction (*e.g.*, CO_2_ → *HCOO → *HCOOH) with a fixed free energy difference between the initial and final states, *U*_OP_ is minimized when *U*_L_ values of the two separate steps are equal. Thus, we can define an overpotential minimum “trough” on which Δ*G*[CO_2_ → *HCOO] equals Δ*G*[HCOO → *HCOOH]. Since the desorption of *HCOOH is disfavored if *G*[*HCOOH] < 1.31 eV,^39^ the gray area in Fig. 2a is excluded from the overpotential trough. We identify 14 BMEs near the trough (Table S3†) and they all favor desorbed *HCOOH as the final product. Next, in Fig. 2b we compare the overall overpotential for HCOOH production *versus* that for the HER on the 14 BMEs. It is found that although all these BMEs are more active toward the CO_2_RR than the HER in the first hydrogenation step (CO_2_ → *HCOO), some of them are less active in the subsequent reduction of *HCOO. More specifically, three Cu based BMEs (Cu^ML^/Ru, Cu^ML^/Ir, and Cu^ML^/Os) are inferior HCOOH catalysts because their *U*_L_ values for the second hydrogenation step (HCOO* → HCOOH) are higher than the *U*_OP_ for the HER (Fig. 2b). The remaining 11 BMEs, Ag^ML^/Zr, Ag^ML^/Nb, Ag^ML^/Hf, Ag^ML^/Ti, Au^ML^/Ta, Au^ML^/Zr, Au^ML^/Nb, Pd^ML^/Ta, Au^ML^/Hf, Ag^ML^/Mo, and Pd^ML^/V, are predicted as superior HCOOH catalysts with a suppressed HER. Note that the calculated overpotentials for HCOOH production on the 11 candidates (0.22–0.36 V *vs.* RHE) are comparable to those found in the most active HCOOH catalysts.^42^ We note that other single- or multi-carbon products such as CO, CH_4_, and C_2_H_4_ cannot be produced on the BMEs due to the absence of the *CO intermediate in the *HCOO pathway. The selective production of HCOOH (as opposed to CO) on the BMEs is due to the preferential binding of O over C on the MLs (Table S4†).

**Fig. 3:**
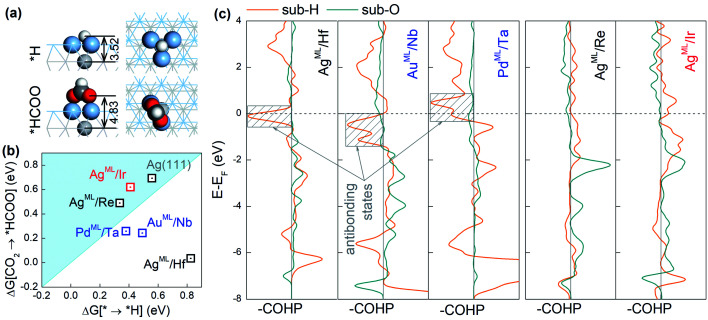
(a) The adsorption structures for *H and *HCOO on Ag^ML^/Hf. The light gray, blue gray, dark gray, red, and white spheres represent Hf, Ag, C, O, and H atoms, respectively. (b) Changes in free energy for HCOO* formation against that for H* formation on selected BMEs. (c) Projected crystal orbital Hamilton population (COHP) for *H–substrate and O–substrate (in *HCOO) interactions in selected BMEs. The contributions of s, p, and d orbitals are included. The bonding and antibonding states are shown on the right and the left of the vertical zero line, respectively. The horizontal dashed line indicates the Fermi level.

The adsorption configurations of *H and *HCOO on the BMEs, taking Ag^ML^/Hf as an example, are shown in Fig. 3a. It is found that *H binds to a threefold hollow ML site and in contrast, the two O atoms in *HCOO bind to the top ML sites. As a result, the bond length (3.52 Å) between *H and the substrate metal atoms is much shorter than the bond length (4.83 Å) between O and the substrate metal atoms (Fig. 3a). This suggests that the substrate atoms underneath the ML may interact with *H much more strongly than with *HCOO. That is to say, *H bonds to both the ML and the substrate atoms while HCOO* only bonds to the ML atoms. In other words, although on the same ML surface, the two intermediates *H and *HCOO “see” different chemistries, which enables them to circumvent the scaling relationship. To support our claim, we will first confirm that *H can indeed interact strongly with the substrate atoms. As shown in Fig. S2,† there is a significant charge rearrangement (both charge accumulation and deficit) around the subsurface Hf atoms in Ag^ML^/Hf. In contrast, such a rearrangement is negligible on a pure Ag surface. This confirms that *H can indeed interact strongly with the subsurface Hf atoms, but not so with the subsurface Ag atoms. This is the reason why the scaling relationship holds for the pure substrates, but not for BMEs.

Next, we compute the Crystal Orbital Hamilton Population (COHP)^43,44^ to shed light on the nature of *H–substrate interactions. COHP projects the band structure energy into orbital–pair interactions and can provide relative contributions of bonding, non-bonding, and antibonding states for a given bond as a function of the energy. In convention, a negative COHP represents bonding interaction. In Fig. 3c, –COHP is plotted instead so that the bonding (antibonding) states are shown on the right (left) side of the vertical zero COHP line. To correlate COHP with the CO_2_RR on BMEs, we choose three superior CO_2_RR catalysts (Ag^ML^/Hf, Au^ML^/Nb, and Pd^ML^/Ta) below the iso-energy line (in Fig. 2a) and two inferior CO_2_RR catalysts (Ag^ML^/Re and Ag^ML^/Ir) above the iso-energy line for comparison. As shown in Fig. 3c, all three superior BME catalysts feature prominent contributions from antibonding states (shaded boxes) near the Fermi level for *H–substrate bonding. In sharp contrast, no such antibonding states exist in the two inferior BME catalysts. Importantly, we find that the strong *H–substrate antibonding contributions in the superior CO_2_RR catalysts are from d orbitals of the substrate metals (Fig. S3†). According to the d-band theory,^45^ the adsorption properties on transition metals are governed by the filling of their antibonding states. Strong bonding occurs if the antibonding states are shifted up in energy and emptied. Conversely, as in the case of the three superior catalysts, the filling of *H–substrate antibonding states weakens the adsorption of *H and thus suppresses the HER. In contrast, there is negligible antibonding interaction between *H and the substrates in the inferior catalysts, thus the adsorption of *H remains too strong on these catalysts. On the other hand, the O–substrate bonding does not feature the strong antibonding contribution in any BME examined here, which implies that adsorption of *HCOO on the BMEs cannot be substantially weakened by the substrates. These results suggest that the antibonding interaction between *H and the substrate beneath the ML is responsible for the decoupling of *H and *HCOO adsorption on the BMEs and for the circumvention of the scaling relationship. Finally, we note that the conclusions drawn here are not confined to the BMEs, and they are applicable to other metallic nanostructures, such as transition metal near-surface alloys and bimetallenes,^39,40^ with ultrathin top layers on which *H–substrate antibonding interaction can also suppress the HER.

**Fig. 4:**
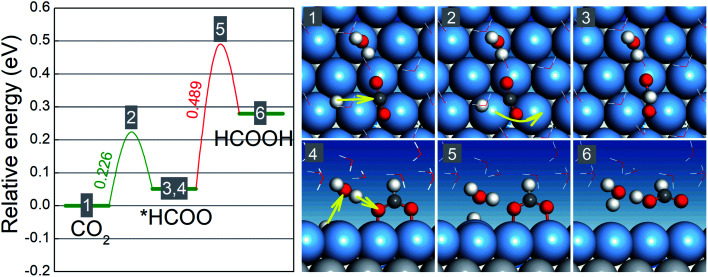
Schematic free energy diagram and optimized atomic structures of the initial states (1,4), transition states (2,5), and final states (3,6) for the hydrogenation of CO_2_ to *HCOO and the hydrogenation of *HCOO to HCOOH on the Ag^ML^/Ti surface at zero voltage. The light gray, blue gray, dark gray, red, and white spheres represent Ti, Ag, C, O, and H atoms, respectively.

Our preceding discussion is focused on the free energy differences between the initial and final states in the two reaction steps of the CO_2_RR and HER, but the relevant activation energy barriers have not been taken into consideration thus far. In the following, we pay close attention to the CO_2_RR mechanism on BMEs by computing the relevant activation energy barriers explicitly.^46,47^ To this end, we choose Ag^ML^/Ti as an example because its (Ag) lattice constant is close to that of Pt (111) on which the ice-like water bilayer structure is known and can be adopted to capture the solvation effect.^41^ As shown in Fig. 4, the activation of the first hydrogenation step (CO_2_ → *HCOO) involves a proton transfer to the carbon atom and simultaneous bending and rotation of CO_2_ to form a C–H bond. The free energy barrier for this step is 0.23 eV, well below the free energy increases for the branched reduction of CO_2_ to *COOH (0.79 eV) and the formation of *H (0.66 eV) (Table S3†). Note that the activation barrier for *COOH and *H formation should always be higher than the corresponding free energy increases although the former is not explicitly calculated here. Thus, for the first hydrogenation step, Ag^ML^/Ti is predicted to selectively produce *HCOO, as opposed to *COOH or *H. In the second hydrogenation step (*HCOO → HCOOH), a Grotthuss mechanism is identified: a proton is transferred to a water molecule, which concurrently shuttles another proton to the O atom in *HCOO to form HCOOH. Since *HCOO is anchored on the surface *via* the O atoms, the reduction is slightly hindered due to the surface–O bonding. The energy barrier for the second step, as a result, is calculated as 0.49 eV, which is nonetheless lower than the free energy increases for *COOH and *H formation. In other words, in the second hydrogenation step, Ag^ML^/Ti can selectively produce HCOOH, as opposed to *COOH and *H. Therefore, by taking into consideration the free energy barriers, we confirm our previous results − which were based on the free energy differences − that Ag^ML^/Ti is an active and selective CO_2_RR catalyst. Additionally, we note that the high energy barrier for the *HCOO → HCOOH step could be lowered when the adsorption of *HCOO is weakened. Hence, BMEs above the overpotential trough in Fig. 2a are predicted to have weaker binding with *HCOO and are more desirable as HCOOH catalysts.

**Fig. 5:**
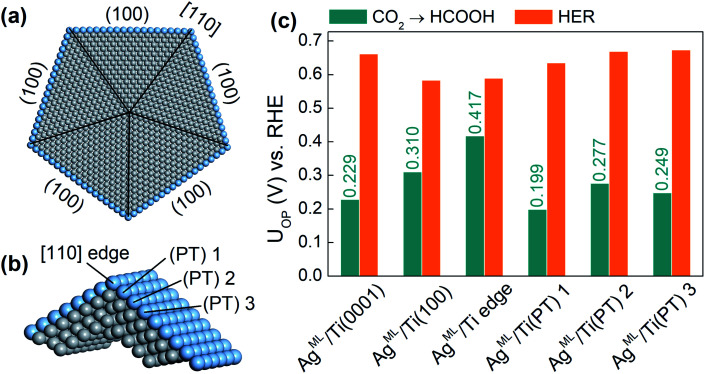
(a) The atomic structure for the ML coated penta-twinned nanowire (PTNW) of a diameter of 8.5 nm. (b) The supercell for the PTMW used in the DFT calculations. (c) Column chart comparison of *U*_OP_ for HCOOH production and the HER on selected surface sites.

Finally, we extend our study to an ML-coated penta-twinned nanowire (PTNW) to establish the generality of our results. Specifically, we take Ag^ML^/Ti as a reference and construct an Ag ML coated penta-twinned Ti nanowire with a circumscribed circle diameter of 8.5 nm (Fig. 5a). PTNW is chosen as the core because of its stability and ultrahigh mechanical strength.^48^ The penta-twinned structure is based on an fcc lattice with five twin boundaries (black lines in Fig. 5a), five [110] edges and five (100) facets. To examine the CO_2_RR on different nanowire surface sites, two slab models are considered. A Ag^ML^/Ti (100) slab model is constructed from an fcc Ti lattice to model the surface sites farther away from the [110] edge. For the surface sites close to the [110] edge, a slab model including two intersecting (100) facets is constructed based on a relaxed penta-twinned structure from a large-scale molecular mechanics simulation (Fig. 5b). In Fig. 5c, we compare the overpotentials for HCOOH production with those for the HER on selected surface sites of PTNW. We find that all these surface sites are more active toward HCOOH production than the HER, in support of our previous conclusion that the scaling relationship between *H and *HCOO adsorption can be circumvented on ML-coated nanostructures. Note that the overpotentials for HCOOH production are quite sensitive to the change in the binding sites on PTNW, which is attributed to the unique twin boundary structure and surface strains. In fact, some active sites, such as Ag^ML^/Ti(PT) 1 on the PTNW surface (Fig. 5c), could yield an overpotential for HCOOH production as low as 0.20 V, thanks to ∼0.8% uniaxial compression on the site.

The present work provides a concrete example of “multisite functionalization” which has been envisioned as a promising strategy to circumvent the scaling relationships.^13^ Although our work focuses on a specific reaction (CO_2_RR) on a specific class of catalysts (BMEs), the general idea is applicable to other metals (*e.g.*, intermetallic compounds) and alloys. However, the complex electronic structures of alloys may obscure the analysis of *H–substrate interaction where the contributions of various alloy elements must be taken into account. In contrast, focusing on pure transition metals in BMEs enables us to illustrate the underlying physics more clearly. To provide more insight on how *H–substrate interaction may influence the CO_2_RR on the BMEs, we further compare three BMEs with the same substrate (Nb), including Ag^ML^/Nb, Au^ML^/Nb, and Pd^ML^/Nb; the first two were predicted as superior catalysts while the third one as an inferior catalyst for the CO_2_RR. COHP is calculated for the three BMEs and the result is shown in Fig. S4.† We find that *H–substrate antibonding interaction depends sensitively on the *H–substrate distance. The *H–substrate distances are nearly the same on Ag^ML^/Nb and Au^ML^/Nb, and *H adsorption is weakened on both BMEs thanks to their filled antibonding states. In contrast, the *H–substrate distance on Pd^ML^/Nb is significantly shortened, as compared with the other two BMEs. The reduced distance shifts the antibonding states up in energy and empties these antibonding states. As a result, *H adsorption remains strong on Pd ML. These results highlight the importance of *H–substrate distance and explain why Ag^ML^/Nb and Au^ML^/Nb are superior catalysts while Pd^ML^/Nb is not.

## Conclusions

In summary, we predict that transition metal MLs placed on extended hcp (0001), fcc (111), and bcc (110) metals (BMEs) are promising electrocatalysts for the CO_2_RR based on systematic DFT calculations. We have identified 11 Ag, Au, and Pd based BMEs (Zr, Nb, Hf, Ti, Ta, Mo, and V as the substrates) which are highly active and selective toward the production of HCOOH. The competing HER is suppressed on all these BMEs, which can be attributed to H*–subsurface (or substrate) antibonding interaction. Relative to *HCOO adsorption on the BMEs, *H adsorption is drastically weakened by the antibonding interaction and as a result, the scaling relationship dictating the competition of the CO_2_RR and HER is circumvented. Taking Ag coated Ti penta-twinned nanowire (PTNW) as an example, we further demonstrate that the correlation of *H and *HCOO adsorption on PTNWs can be broken which render the PTNWs as highly active and selective catalysts for the CO_2_RR. The free energy barriers for the hydrogenation of CO_2_ to HCOOH are also calculated to elucidate the reaction mechanism and validate our conclusions. The antibonding adsorbate–substrate interaction highlighted in this work is also expected to play important roles in other nanocatalysts as a means of circumventing undesirable scaling relationships.

## Data availability

The data that support the findings of this study are available from the corresponding author upon reasonable request.

## Author contributions

ZZ and GL conceived the project. ZZ performed calculations. ZZ and GL analyzed the data and wrote the paper.

## Conflicts of interest

There are no conflicts to declare.

## Supplementary Material

SC-013-D2SC00135G-s001
