# Supplementary material for: Circumventing the scaling relationship on bimetallic monolayer electrocatalysts for selective CO2 reduction
Source: Chem Sci. 2022 Mar 14;13(13):3880–7. doi: 10.1039/d2sc00135g (PMC8966713; doi:10.1039/d2sc00135g)
Supplement: SC-013-D2SC00135G-s001 [file SC-013-D2SC00135G-s001.pdf]

Electronic Supplementary Material (ESI) for Chemical Science.
This journal is © The Royal Society of Chemistry 2022

Supporting Information

Circumventing Scaling Relationship on Bimetallic Monolayer Electrocatalysts for Selective CO_2 Reduction

Zhonglong Zhao[a] and Gang Lu*[b]

[a]*School of Physical Science and Technology, Inner Mongolia University, Hohhot 010021, China*
[b]*Department of Physics and Astronomy, California State University Northridge, California 91330, United States. Email: ganglu@csun.edu*

COMPUTATIONAL DETAILS

a. Free energy, limiting potential, overpotential, and reaction pathways for CO_2RR

The reaction free energy of the intermediates is derived from the binding energy (E_B) at 18.5 °C by including the zero-point energy (ZPE), heat capacity (C_P) and entropy (-TS) corrections:[1]

$$G = E_B + ZPE + \int C_P dT - TS \quad (1)$$

All the free energy corrections are calculated based on the molecular vibration analysis and assuming that the changes in the vibrations of the surface caused by the intermediate are minimal. We applied approximate solvation corrections to the reaction intermediates proposed by Peterson et al.[2] The binding energy of an intermediate is calculated as:

$$E_B[C_xH_yO_z] = E[C_xH_yO_z] - E_{slab} - xE_C - yE_H - zE_O \quad (2)$$

Where $E[C_xH_yO_z]$ and E_{slab} denote the total energy of the system with and without the intermediate, respectively. E_C, E_H, and E_O are the total energy of one atom in grapheme, gaseous hydrogen, and the difference between H_2O and H_2, respectively.

According to the computational hydrogen electrode (CHE) model, the limiting potential (U_L) for the reaction step, for example, $CO_2 \rightarrow$ *HCOO, is defined as the change of the free energies between *HCOO and CO_2, in addition to the chemical potential of a proton-electron pair $\mu(H^+ + e^-)$, calculated as half of the free energy of gas-phase H_2 at zero applied voltage:

$$U_L = -\frac{G[HCOO] - G[CO_2] - \frac{1}{2}G[H_{2g}](U = 0V_{RHE})}{e} \quad (3)$$

The free energies of non-adsorbed species such as CO_2 and HCOOH are taken from previous work.[2] Note that to correct the inconsistency between the theoretical and experimental gas-phase reaction enthalpies, +0.45 eV is added to the energy of CO_2 and HCOOH, as also proposed in previous work.[2]

The following reaction pathways for CO_2 hydrogenation have been considered in this work:

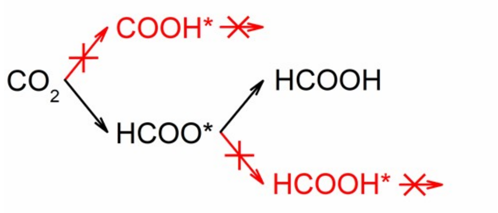

As discussed in the main paper, the reduction of CO_2 into *COOH is disfavored on the proposed BMEs and CO_2 is only expected to be reduced into *HCOO. Thus, the reactions after *COOH

are excluded. In addition, *CO cannot be reduced from *HCOO because the reduction requires breaking of a C-O bond and a C-H bond in *HCOO, which is energetically unfavorable. Thus, the reduction of *HCOO to *CO and other intermediates after *CO is also excluded. Furthermore, because the desorption of *HCOOH is exothermic on the proposed BMEs, *HCOOH cannot be reduced further and is the final product. Hence, no products other than HCOOH can be produced on the proposed BMEs. Based on the two-step reaction ($CO_2 \rightarrow$ *HCOO $\rightarrow$ HCOOH), the more negative U_L among the two steps is defined as the reaction overpotential U_{OP} in this work.

b. Activation barrier calculations

The activation barrier for the hydrogenation reaction is calculated based on the model proposed by Nie et al.[3-4] In the model, the activation barrier for an elementary electrochemical reaction ($A^* + H^+ + e^- \rightarrow AH^*$) is derived from analogous surface hydrogenation reaction ($A^* + H^* \rightarrow AH^*$). The activation barrier as a function of the electrode potential U is calculated as:

$$E_{act}(U) = E_{act}^0 + \beta'(U - U^0) \quad (3)$$

where E_{act}^0 is the reaction barrier calculated from DFT plus the ZPE correction. U^0 is set so that the chemical potential of the adsorbed H* is equal to that of a proton-electron pair. β' is an effective symmetry factor calculated by:

$$\beta' = 0.5 + (\mu_{TS} - \mu_{reactant})/3 \quad (4)$$

where $\mu_{TS} - \mu_{reactant}$ represents the variation in the surface dipole moments between the reactant and the transition state.

c. Water-Assisted reaction model

In the activation barrier calculations, we considered the presence of one water molecule below the H-down ice-like water bilayer to assist the hydrogenation reactions. More specifically, water can assist the reaction in two manners: (1) the surface proton is transferred directly to the adsorbate, assisted by the hydrogen bond between the water molecule and the adsorbate; (2) the surface proton is transferred to the water molecule, which concurrently shuttles another proton to the adsorbate, analogous to the Grotthuss mechanism.

We note that the above water-assisted activation barrier calculation model was able to reproduce experimentally identified CO_2RR species on Cu, and also predicted a correct methanol product for CH_2O reduction on Cu.[3-4] Besides, the model was also used to examine C_2 product selectivity on Cu(100) for CO_2RR,[5] to predict Cu monolayer catalysts and bimetallic alloys for

CO_2RR,[6-7] to study binary metal catalyst for electrochemical nitrogen reduction reaction,[8] to design $Au_{22}(L^8)_6$ nano-clusters for oxygen reduction reaction,[9] and to predict bimetallenes for selective CO_2RR to HCOOH.[10]

Table S1. The calculated formation energy (E_f) and segregation energy (E_{seg}) of the stable BMEs. Negative E_f and positive E_{seg} values indicate that the catalyst is stable.

ML/hcp(0001)	E_f	E_{seg}	ML/fcc(111)	E_f	E_{seg}	ML/bcc(110)	E_f	E_{seg}
Ag/Ti	-0.351	0.951	Ag/Rh	-0.349	1.198	Ag/V	-0.195	0.255
Cu/Ti	-0.489	0.645	Au/Rh	-0.317	0.605	Cu/V	-0.327	0.826
Pd/Ti	-0.731	0.199	Cu/Rh	-0.451	0.448	Pd/V	-0.525	0.717
Ag/Zr	-0.424	1.201	Pd/Rh	-0.489	0.570	Ag/Nb	-0.245	1.963
Au/Zr	-0.579	0.420	Pt/Rh	-0.496	0.421	Au/Nb	-0.312	0.830
Cu/Zr	-0.570	0.081	Ag/Pd	-0.534	0.141	Cu/Nb	-0.388	0.770
Ag/Ru	-0.194	2.381	Pt/Ag	-0.787	0.046	Pd/Nb	-0.623	1.141
Au/Ru	-0.174	1.702	Ag/Ir	-0.288	1.771	Ag/Mo	-0.096	1.902
Cu/Ru	-0.307	1.264	Au/Ir	-0.248	1.272	Au/Mo	-0.106	1.055
Pd/Ru	-0.390	1.344	Cu/Ir	-0.421	0.775	Cu/Mo	-0.247	1.093
Pt/Ru	-0.403	0.879	Pd/Ir	-0.472	0.767	Pd/Mo	-0.374	0.950
Ag/Hf	-0.332	1.497	Pt/Ir	-0.472	0.620	Ag/Ta	-0.148	1.900
Au/Hf	-0.487	0.686	Ag/Pt	-0.522	0.668	Au/Ta	-0.226	0.843
Cu/Hf	-0.489	0.543	Au/Pt	-0.466	0.534	Cu/Ta	-0.312	0.888
Ag/Re	-0.001	2.200				Pd/Ta	-0.574	1.207
Cu/Re	-0.135	0.709				Au/W	-0.000	1.708
Pd/Re	-0.265	0.697				Cu/W	-0.162	1.682
Ag/Os	-0.118	3.371				Pd/W	-0.302	1.558
Au/Os	-0.091	2.747				Pt/W	-0.357	0.306
Cu/Os	-0.252	1.997						
Pd/Os	-0.334	1.623						
Pt/Os	-0.347	1.159						

Table S2. Standard dissolution potential U_{diss}(bulk) of the bulk metal of the MLs, formation energy E_f of the BMEs, number of transferred electrons during the dissolution (N_e), and the dissolution potential U_{diss} of the proposed BMEs. The proposed catalysts with a positive U_{diss} (V vs SHE) are regarded as electrochemically stable under acidic conditions.

ML/substrate	U_{diss}(bulk) (V)	E_f (eV)	N_e	U_{diss} (V)
Ag/Ti(0001)	0.80	0.264	1	0.536
Ag/Zr(0001)	0.80	0.191	1	0.609
Au/Zr(0001)	1.50	0.037	3	1.488
Ag/Hf(0001)	0.80	0.283	1	0.517
Au/Hf(0001)	1.50	0.129	3	1.457
Pd/V(110)	0.95	0.090	2	0.905
Ag/Nb(110)	0.80	0.370	1	0.430
Au/Nb(110)	1.50	0.304	3	1.399
Ag/Mo(110)	0.80	0.520	1	0.280
Au/Ta(110)	1.50	0.390	3	1.370
Pd/Ta(110)	0.95	0.042	2	0.929

Table S3. Free energy changes for the formation of *H, the reduction of CO_2 to *HCOO and *COOH, the reduction of *HCOO to HCOOH*, and desorption of *HCOOH on the proposed BMEs.

ML/substrate	* → *H	CO_2 → *HCOO	CO_2 → *COOH	*HCOO → HCOOH*	*HCOOH → HCOOH
Ag/Ti(0001)	0.662	0.051	0.787	0.290	-0.061
Cu/Ti(0001)	0.385	-0.409	0.478	0.675	0.014
Ag/Zr(0001)	0.705	-0.082	0.701	0.435	-0.073
Au/Zr(0001)	0.905	0.225	0.827	0.148	-0.093
Cu/Ru(0001)	0.077	-0.045	0.481	0.364	-0.039
Ag/Hf(0001)	0.825	0.035	0.824	0.318	-0.073
Au/Hf(0001)	0.990	0.296	0.930	0.078	-0.094
Cu/Re(0001)	-0.221	-0.440	0.070	0.552	0.168
Cu/Os(0001)	0.113	0.014	0.521	0.259	0.007
Cu/Ir(111)	0.067	0.002	0.453	0.274	0.004
Cu/V(110)	0.021	-0.436	0.210	0.647	0.069
Pd/V(110)	0.373	0.355	0.787	-0.149	0.074
Ag/Nb(110)	0.334	-0.042	0.632	0.365	-0.043
Au/Nb(110)	0.492	0.243	0.655	0.110	-0.073
Cu/Nb(110)	0.006	-0.712	-0.004	0.865	0.127
Ag/Mo(110)	0.408	0.354	0.963	0.015	-0.089
Cu/Mo(110)	0.040	-0.276	0.312	0.472	0.084
Ag/Ta(110)	0.285	-0.150	0.526	0.442	-0.012
Au/Ta(110)	0.498	0.063	0.561	0.273	-0.056
Cu/Ta(110)	0.004	-0.752	-0.026	0.914	0.118
Pd/Ta(110)	0.379	0.259	0.807	0.010	0.011
Cu/W(110)	-0.072	-0.342	0.225	0.472	0.150
Pd/W(110)	-0.406	-0.500	-0.094	0.203	0.577

Table S4. The adsorption distance of *COOH and Bader charge transfer from the substrate to the surface Ag atoms on pure Ag (111) surface and selected Ag based BMEs. The Ag-O distance is reduced on the BMEs compared with that on Ag(111) surface. In contrast, the Ag-C distance is either the same or slightly longer than that on Ag(111).

Catalysts	Ag-C distance (Å)	Ag-O distance (Å)	Charge transfer/Ag atom
Ag (111)	2.176	2.551	none
Ag/Ti(0001)	2.183	2.471	0.327*e*
Ag/Zr(0001)	2.173	2.493	0.388e
Ag/Hf(0001)	2.213	2.449	0.393*e*
Ag/Nb(110)	2.199	2.520	0.266*e*
Ag/Mo(110)	2.210	2.458	0.140*e*

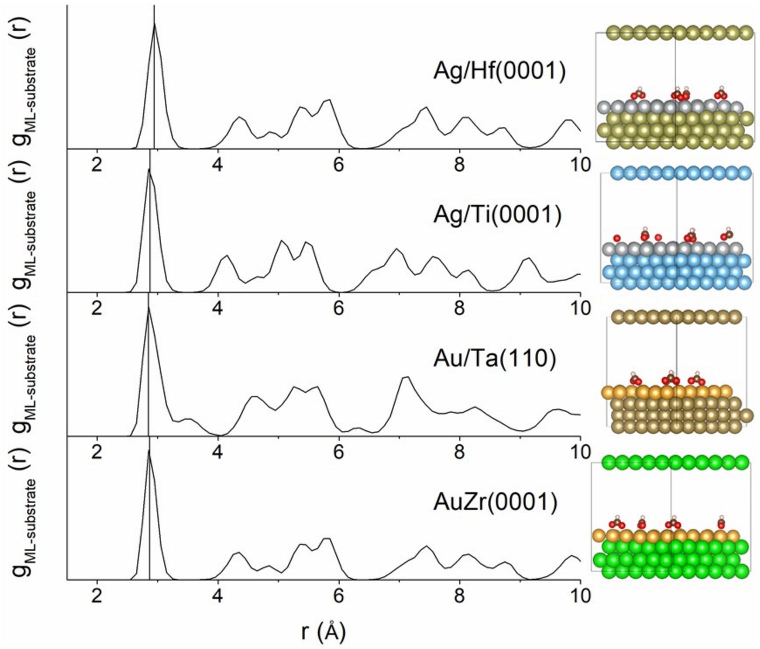

Figure S1 Radial distribution function and crystal structure of the selected BMEs at 300 K in the presence of HCOO* on the surface after a 4 ps MD simulation. The vertical lines indicate the equilibrium nearest-neighbor (NN) distance at 0 K. The proposed BMEs are found to be stable because of the negligible changes in the NN distance between 0 K and 300 K.

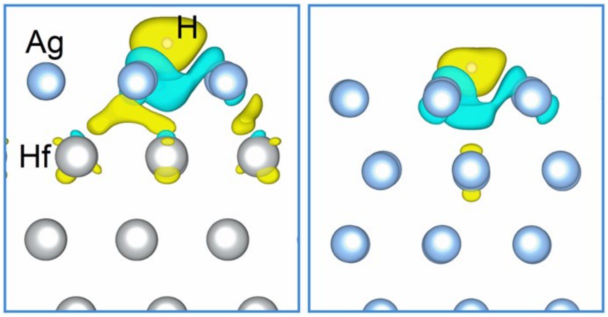

Figure S2 The adsorption structure and differential charge density isosurfaces for *H on Ag^{ML}/Hf and pure Ag surfaces. Cyan and yellow isosurfaces correspond to the charge density contour of -0.01 and +0.01 e $Å^{-3}$. Light gray, blue gray, and white spheres represent Hf, Ag, and H atoms, respectively.

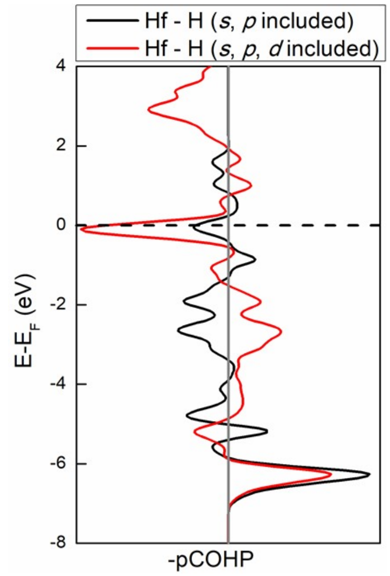

Figure S3 Projected crystal orbital Hamilton population (pCOHP) for the subsurface Hf - H interaction on Ag^{ML}/Hf surface calculated with (red) and without (black) the Hf *d* orbitals. Bonding and antibonding states are shown on the right and left, respectively. The horizontal dashed lines indicate the Fermi level.

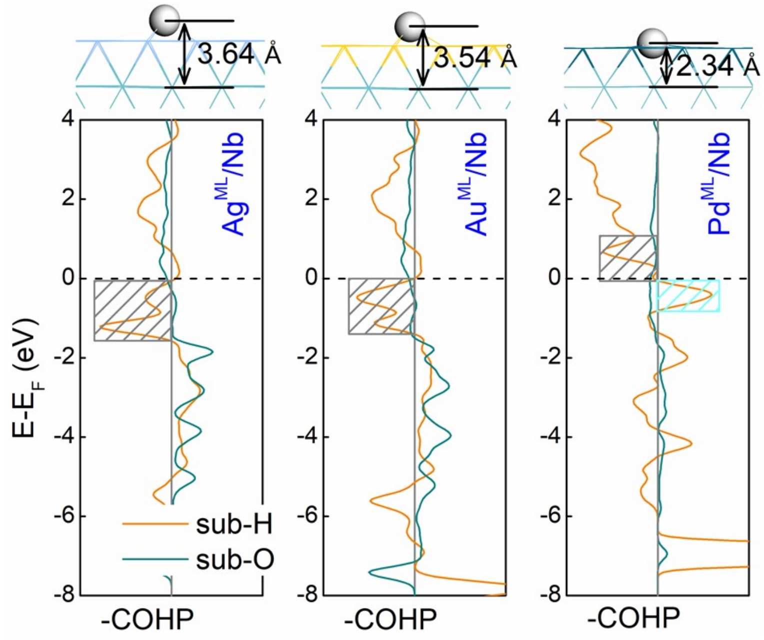

Figure S4 The adsorption structures and projected crystal orbital Hamilton population (COHP) for *H-substrate and O-substrate (in *HCOO) interactions in the three BMEs. The contributions of *s*, *p*, and *d* orbitals are included. The antibonding and bonding states are shown on the left and the right of the vertical zero line. The horizontal dashed line indicates the Fermi level.

References

(1) Cramer, C. J. *Essentials of Computational Chemistry: Theories and Models*. 2nd ed.; Wiley & Sons: Chichester, U.K., **2004**.

(2) Peterson, A. A.; Abild-Pedersen, F.; Studt, F.; Rossmeisl, J.; Norskov, J. K. How Copper Catalyzes the Electroreduction of Carbon Dioxide into Hydrocarbon fuels. *Energy Environ. Sci.* **2010**, *3*, 1311-1315.

(3) Nie, X.; Esopi, M. R.; Janik, M. J.; Asthagiri, A. Selectivity of CO_2 Reduction on Copper Electrodes: The Role of the Kinetics of Elementary Steps. *Angew. Chem., Int. Ed.* **2013**, *52*, 2459-2462

(4) Nie, X.; Luo, W.; Janik, M. J.; Asthagiri, A. Reaction Mechanisms of CO_2 Electrochemical Reduction on Cu(111) Determined with Density Functional Theory. *J. Catal.* **2014**, *312*, 108-122.

(5) Luo, W.; Nie, X.; Janik, M. J.; Asthagiri, A. Facet Dependence of CO_2 Reduction Paths on Cu Electrodes. *ACS Catal.* **2016,** *6*, 219-229.

(6) Mandal, S. C.; Rawat, K. S.; Garg, P.; Pathak, B. Hexagonal Cu(111) Monolayers for Selective CO_2 Hydrogenation to CH_3OH: Insights from Density Functional Theory. *ACS Applied Nano Materials* **2019,** *2*, 7686-7695.

(7) Wu, N.; Xiao, L.; Zhuang, L. Theoretical Search for Novel Au or Ag Bimetallic Alloys Capable of Transforming CO_2 into Hydrocarbons. *J. Mater. Chem. A* **2019,** *7*, 20567-20573.

(8) Liu, X.; Jiao, Y.; Zheng, Y.; Qiao, S.-Z. Isolated Boron Sites for Electroreduction of Dinitrogen to Ammonia. *ACS Catal.* **2020,** *10*, 1847-1854.

(9) Deng, C.; Li, F.; Tang, Q. Electrocatalytic Oxygen Reduction Reaction over the $Au_{22}(L^8)_6$ Nanocluster with Promising Activity: A DFT Study. *J. Phys. Chem. C* **2019,** *123*, 27116-27123.

(10) Zhao, Z.; Lu, G. Bimetallenes for Selective Electrocatalytic Conversion of CO_2: A First-Principles Study. *J. Mater. Chem. A* **2020,** *8*, 12457-12462.
